# Postantifungal Effect of Antifungal Drugs against *Candida*: What Do We Know and How Can We Apply This Knowledge in the Clinical Setting?

**DOI:** 10.3390/jof8070727

**Published:** 2022-07-12

**Authors:** Nerea Jauregizar, Guillermo Quindós, Sandra Gil-Alonso, Elena Suárez, Elena Sevillano, Elena Eraso

**Affiliations:** 1 Department of Pharmacology, Faculty of Medicine and Nursing, University of the Basque Country (UPV/EHU), 48940 Bilbao, Spain; elena.suarez@ehu.eus; 2 Department of Immunology, Microbiology and Parasitology, Faculty of Medicine and Nursing, University of the Basque Country (UPV/EHU), 48940 Bilbao, Spain; guillermo.quindos@ehu.eus (G.Q.); sandra.gil@ehu.eus (S.G.-A.); elena.sevillano@ehu.eus (E.S.); elena.eraso@ehu.eus (E.E.)

**Keywords:** postantifungal effect, antifungal therapy, candidiasis, polyenes, azoles, echinocandins, 5-fluorocytosine, dosing regimen

## Abstract

The study of the pharmacological properties of an antifungal agent integrates the drug pharmacokinetics, the fungal growth inhibition, the fungicidal effect and the postantifungal activity, laying the basis to guide optimal dosing regimen selection. The current manuscript reviews concepts regarding the postantifungal effect (PAFE) of the main classes of drugs used to treat *Candida* infections or candidiasis. The existence of PAFE and its magnitude are highly dependent on both the fungal species and the class of the antifungal agent. Therefore, the aim of this article was to compile the information described in the literature concerning the PAFE of polyenes, azoles and echinocandins against the *Candida* species of medical interest. In addition, the mechanisms involved in these phenomena, methods of study, and finally, the clinical applicability of these studies relating to the design of dosing regimens were reviewed and discussed. Additionally, different factors that could determine the variability in the PAFE were described. Most PAFE studies were conducted in vitro, and a scarcity of PAFE studies in animal models was observed. It can be stated that the echinocandins cause the most prolonged PAFE, followed by polyenes and azoles. In the case of the triazoles, it is worth noting the inconsistency found between in vitro and in vivo studies.

## 1. Introduction

The therapeutic approach of systemic candidiasis is focused on clinical, microbiological and pharmacological criteria, including the immune status of the patient, specific characteristics of the infectious disease and the pharmacokinetic (PK) and pharmacodynamic (PD) features of the antifungal agent [1]. In contrast to antibacterial drugs, the toolbox for dealing with invasive mycoses is not so varied. There are only four main classes of compounds that can have a systemic antifungal effect: the polyenes (amphotericin B), the azoles that comprise the largest number of drugs (fluconazole, itraconazole, posaconazole, voriconazole and isavuconazole), the echinocandins (anidulafungin, caspofungin and micafungin) and 5-fluorocytosine.

Due to the relatively scarce options to treat invasive fungal infections and the emergence of resistant strains and new fungal species, such as *Candida auris*, there is an urgent need to enhance the therapeutic options. There are different strategies available to combat the emergence of antimicrobial resistance and the scarcity of therapeutic options, such as the development of novel agents, drug repositioning and the more effective use of existing drugs. In addition, the development of a new drug is a process that consumes significant resources in terms of time and cost. Moreover, without financial incentives, many pharmaceutical companies would not develop or market these drugs. In this scenario, it is absolutely essential to focus antimicrobial therapeutics on the best knowledge of existing treatments and use this knowledge to design better antimicrobial treatment protocols. In this regard, by optimizing the dosing of currently available antimicrobials, the clinical efficacy is enhanced. The knowledge of the PK/PD or the study of the time evolution of antimicrobial therapy improves the efficacy of antimicrobial drugs and facilitates the selection of the optimal therapy for drug resistant infections.

There are two main characteristics that determine the time course of antifungal effect, and consequently, the selection of dose interval. Firstly, how the increasing drug concentrations, whose evolution varies depending on drug PK properties as half-life, impact on the rate and extent of the fungal killing or growth inhibition. Secondly, the absence or presence of antifungal effect which persists after drug concentrations fall below the minimum inhibitory concentration (MIC). In fact, the length of the anti-infective action after a drug clears from the site of infection impacts both on the dose interval choice and the outcome of the infectious disease [2]. Whereas antifungal drugs that exhibit long postantifungal effect (PAFE) may be given less frequently, those drugs with short PAFE may require frequent administration [3].

PAFE is the persistent activity of the drug once the isolates are exposed to it, despite the absence of measurable concentrations of the drug in contact with the fungus. It is determined as the continuation of the drug effect of suppression of fungal growth after it has been removed from the fungal suspension or the infection site [3]. It is known that the existence of PAFE depends, among other factors, on both the fungal species and the class of the antifungal drug. This review examines the post-drug exposure effects induced by polyenes (amphotericin B and nystatin), azoles, echinocandins and 5-fluorocytosine against the main *Candida* species, including the most prevalent in human disease as well as emerging species. In addition, the review aims to compile information regarding the observed or suggested mechanisms involved in these phenomena, the methods to study the PAFE and, finally, the clinical applicability of these studies relating to the design of dosing regimens.

**Figure 1:**
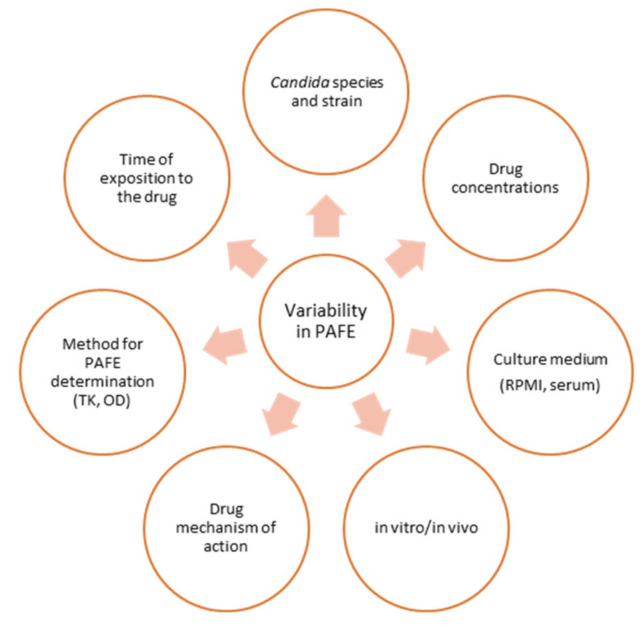
Different factors that determine the variability in PAFE. TK: Time-kill; OD: Optical density.

It should be highlighted that the determination of the PAFE and, therefore, its results and interpretation, are subject to great variability, as summarized in Figure 1. All these factors will be reviewed in this paper and will be taken into account when interpreting the results of the different studies.

## 2. In Vitro and In Vivo Methods for PAFE Study

As PAFE is an important characteristic of antifungal drugs that may influence their PD, it is essential to study this effect both in vitro and in vivo. Factors that may influence the variability of the PAFE include the methodology employed, the drug exposure time, the antifungal class and drug concentrations or fungal species and strains evaluated (Figure 1).

Despite the importance that PAFE may have on the drug PD, few studies have analyzed this effect both in vitro and in vivo.

### 2.1. In Vitro Methods for PAFE Study

There are mainly two methodologies for the in vitro study of PAFE, which are carried out in most of the works reviewed; those based on the measurement of optical density (OD) and those based on the counting of colonies (CC), the latter carried out with killing curves experiments. Chryssanthou et al. [4], on the other hand, analyzed the PAFE employing an automated BacT/Alert system, which is based on the colorimetric detection of CO_2_.

#### 2.1.1. PAFE Determination by Optical Density Measurement

The research group from Kuwait University has been studying the PAFE of different antifungal drugs on *Candida* since 1998. These authors, among others, have measured OD, by using the principle of periodic turbidimetric assessment of growth rates [5,6,7,8,9,10,11,12,13,14].

This methodology follows this protocol: *Candida* strains are subcultured into Sabouraud dextrose agar (SDA) plates at 37 °C for 24 h prior to testing. The starting inoculum is usually 10^6^ *Candida* colony forming units (CFU)/mL in RPMI 1640 (control) and RPMI 1640/drug (test). The concentrations of antifungal drugs used are those corresponding to between two or three times the MIC values, depending on the study [10].

Following 30 or 60 min of exposure, drugs are removed by two or three cycles of dilution using sterile phosphate buffered saline (PBS). After resuspending the cells in PBS and incubating at 37 °C, the optical density of the different samples is measured every 15 min for 5 or 6 h [10,11,12,13,14]. In other studies conducted by these authors, the optical density is measured at 30 min intervals for 8 or 18 h [6,7,9] using a computerized spectrophotometric incubator. All experiments are carried out in triplicate.

##### Data Analysis

PAFE is calculated according to the equation: PAFE = T − C; where T is the time required for the relative OD of the drug-exposed cell suspension to reach the 0.05 absorbance value at 520 nm after drug removal and C is the time required for the relative OD of the drug-free control cell suspension to reach the same absorbance value. Therefore, T − C refers to the time (h) that the effect of drugs against *Candida* lasts after a given exposure [5,6,7,9,10,11,12,13,14].

##### Determination of Carry-Over Effect

The carry-over effect refers to the possibility that the drug has not been effectively removed after the drug washing or removal technique. After removal of the drug by washing with PBS and resuspending the pellets, viable cells from the control experiment and the test are counted to check that the drug has been removed correctly and to exclude any carry-over effect [5,6,7,9,10,11,12,13,14].

#### 2.1.2. PAFE Determination by Colony Counting

From the PK/PD point of view, the determination of PAFE by measuring absorbance provides limited information on the kinetics of drug action. In this sense, the measurement of absorbance at a fixed point provides information at a given time, without taking into account the rate of drug activity (fungicidal or fungistatic) or whether increases in drug concentration can lead to an increase in this rate. These factors are important to determine the PAFE more closely in the in vivo scenario.

The methodology carried out to evaluate the PAFE by colony counting methodology is similar in all studies, with slight differences. The following experiments are carried out to obtain time-kill (TK) curves and PAFE curves [3,15,16,17,18,19,20,21,22,23,24,25,26,27,28,29,30,31,32].

##### Time-Kill Assays

TK experiments are performed to assess whether studied antifungal drugs are fungistatic or fungicidal. For this purpose, *Candida* strains are subcultured into SDA plates from 30–37 °C for between 24 and 48 h prior to testing. The suspensions are prepared in RPMI 1640 medium (RPMI) to achieve a starting inoculum of 10^5^–10^6^ CFU/mL. TK curve assays are carried out on tubes or microtiter plates in RPMI. The drug concentrations are selected according to pharmacological properties, toxicity and on the basis of the MIC values [3,15,16,17,18,19,20,21,22,23,24,25,26,27,28,29,30,31,32].

At predetermined time points (0–48 h), the samples are collected (control and test), serially diluted in PBS and plated into SDA. After incubation of the plates from 35–37 °C for between 24 and 48 h *Candida* colonies are counted. The lower limit of accurate and reproducible detectable CFU varies from 30 to 200 CFU/mL, depending on the study [3,15,16,17,18,19,20,21,22,23,24,25,26,27,28,29,30,31,32,33].

##### PAFE Assays

In most PAFE studies, 10^5^–10^6^ CFU/mL of *Candida* are exposed to the same drug concentrations of the aforementioned TK studies for between 1 and 2 h [3,15,16,17,18,19,20,21,22,23,24,25,26,27,28,29,30,31,32]. Other authors have tested the PAFE at lower exposition times of 5, 15, 30 and 60 min [25].

After antifungal drug exposition, cells are washed three times with PBS; then, the fungal pellet is suspended in RPMI and samples are incubated on tubes or on microtiter plates from 35–37 °C for between 24 and 48 h. At the same predetermined time points as TK, samples are serially diluted in PBS and inoculated into an SDA plate for CFU counting. TK and PAFE assays are performed at least in duplicate [3,15,16,17,18,19,20,21,22,23,24,25,26,27,28,29,30,31,32].

Although in most studies PAFE assays are conducted at between 35 °C and 37 °C, García et al. evaluated the influence of temperature (22 °C, 35 °C and 37 °C) and observed that the PAFE duration of some antifungal drugs increased with temperature [34].

##### Data Analysis

Fungicidal activity is described as a ≥3 log_10_ (99.9%) reduction, and fungistatic activity is defined as a <99.9% reduction in CFU from the starting inoculum size. Plots of averaged colony counts (log_10_ CFU/mL) versus time are constructed and compared against a growth control. In addition, the ratios of the log killing during PAFE experiments to the log killing during time-kill experiments can also be calculated. TK and PAFE experiments are performed simultaneously [3,15,21,23,24,29,30,32].

PAFE is calculated as the difference in time required for the control (in the absence of drug) and treated isolates to grow 1 log_10_ following drug removal using the following equation: PAFE = T − C, where T is the time required for counts in treated cultures to increase by 1 log_10_ unit above that seen following drug removal and C is the time required for counts in control to increase by 1 log_10_ unit above that following the last washing [3,15,16,17,18,19,20,21,22,23,24,25,26,27,28,29,30,31,32].

##### Determination of Carry-Over Effect

It is important to determine the carry-over effect before performing lethality curves. If not assessed, a concentration of antifungal that is carried over with the sample can be considered fungistatic or fungicide. Carry-over is determined by spreading into SDA plates a determinate volume of antifungal drug and counting the CFU, then, the drug is compared with the control plates. Carry-over is considered to be absent if the difference in CFU between the control plate and the plate containing the antifungal drug is <25% [21,23,26,29,30,32].

##### In Vitro PAFE of the Combinations between Antifungal Drugs

The PAFE of drug–drug combinations has not been extensively studied. Oz et al. studied the in vitro PAFE of the combinations of antifungal drugs by colony counting methodology [3]. The evaluation of PAFE and the interpretation was made according to the explanation above.

Scalarone et al. evaluated the in vitro PAFE of flucytosine with fluconazole [35,36] and flucytosine with amphotericin B on *Candida albicans* [37]. The methodology they followed was based on the measurement of the turbidity of tubes above explained, with slight differences in inoculums, employed media and temperature [35,36,37]. The interpretation of the PAFE was made according to the explanation above.

##### In Vitro PAFE in Presence of Serum

Most studies that analyzed PAFE in *Candida* were performed in protein-free RPMI. However, this medium does not reflect in vivo conditions as *Candida* is rarely found in a protein-free environment in vivo [38,39]. Therefore, PD parameters of antifungal drugs measured in RPMI likely do not correctly predict efficacy in the case of highly protein-bound antifungal drugs. After absorption, most drugs bind to plasma proteins. They can also leave the plasma and enter the tissues. Protein binding is relevant because only the unbound drug will be available to exert a pharmacological effect. Tissue distribution of drugs is important as infections affect different tissues [38,39]. The influence of serum on the action of antifungal drugs by TK methodology has been extensively studied [40,41,42,43], however, few studies have analyzed the PAFE of these drugs on *Candida* in the presence of human serum [16,28,31]. The methodology used to evaluate this influence is based on the methods explained above, measuring PAFE in media with and without 50% of human serum. The main conclusion, in this regard, is that PD parameters of antifungal drugs, especially those with high plasma protein binding, cannot be correctly predicted in the presence of RPMI. In general, antifungal agents with high protein binding exhibit reduced activity in the presence of serum compared to RPMI alone [16,28,31,44,45].

So far, the influence of serum on PAFE has only been analyzed on *C. albicans* [16,28,44,45] and recently on four strains of *Candida dubliniensis* and two of *Candida africana* [31].

### 2.2. In Vivo Methods for PAFE Study

The in vitro studies are essential to determine the antimicrobial properties of antifungal agents and to elucidate their mechanisms of action; nevertheless, in vivo studies are indispensable to assess whether antimicrobial activity persists in a complex organism.

The in vivo models most commonly used to study the properties of antimicrobials are mice [46]. In fact, in all in vivo studies in which the PAFE was analyzed, murine models of infection were employed to determinate the PD characteristics of the antifungal drugs against *Candida* [47,48,49,50,51,52].

The in vivo PAFE of anidulafungin, posaconazole and ravuconazole on *C. albicans*, *Candida glabrata* and *Candida tropicalis* has been reported in different studies [47,49,50]. The studied animals were ICR/Swiss specific-pathogen-free female, CD1 male [51] and DBA/2 male mice [52].

#### 2.2.1. Murine Models of Disseminated Candidiasis

In order to carry out these experiments, neutropenic murine disseminated candidiasis models were used.

*Candida* strains were subcultured on SDA 24 h prior to infection. The *Candida* inoculum to create the infection in the neutropenic mice was 10^4^–10^6^ CFU/mL. Disseminated infection was achieved injecting a determinate inoculum via the lateral tail vein from 2–5 h prior to the start of drug therapy [47,49,50,51,52].

#### 2.2.2. In Vivo Time-Kill and PAFE Assays

From 1–2 h after infection with *Candida*, mice were treated with intraperitoneal doses of anidulafungin, posaconazole, ravuconazole [47,49,50], isavuconazole, [51], fluconazole, itraconazole or ketoconazole [52]. Groups of three or four treated and control mice were sacrificed at determinate sampling intervals over a total period of between 3 and 4 days. Kidneys were removed at each time point and processed for the determination of numbers of CFU [47,49,50,51,52]. Warn et al. [51] calculated the duration of the in vivo PAFE with a mathematical model.

##### Data Analysis

The duration of the in vivo PAFE is defined following the equation: PAFE = T − C; where C was the time it took for the organism burden in controls to increase by 1 log_10_ CFU/kidney and T was the value from the amount of time it took organism burdens in the treated animals to increase by 1 log_10_ CFU/kidney after serum drug levels fell below the MIC for the organism [47,49,50,51,52].

## 3. PAFE of Polyenes

Polyenes or polyene macrolides are the oldest family of antifungal drugs since they were introduced in the late 1950s. Their potent antifungal activity is due to interaction with fungal cell membrane sterols. The binding of polyenes to ergosterol creates channels in the membrane leading to the leakage of cellular contents through the pores formed. This negatively affects membrane permeability and fluidity, as well as other cellular functions, and leads to cell collapse [53]. Polyenes include several effective antifungal agents, such as amphotericin B and nystatin.

Polyenes have a broad spectrum of antifungal activity that includes yeasts, filamentous fungi and endemic dimorphic fungi. *Candida*, *Cryptococcus*, *Aspergillus*, *Mucor*, *Rhizopus*, *Rhizomucor*, *Histoplasma*, *Coccidioides* and *Blastomyces* are susceptible to polyene action [1,54]. To date, few yeast species present resistance to polyenes, although this resistance is higher among filamentous fungi. Amphotericin B lacks activity against a few filamentous fungi, such as *Aspergillus terreus*, *Fusarium*, *Lomentospora prolificans*, *Scedosporium apiospermum* and *Sporothrix schenckii*. As for yeasts, *Candida lusitaniae*, the *Candida haemulonii* species complex and *C. auris* are considered resistant to this antifungal agent [53].

Amphotericin B is widely used in the treatment of invasive mycoses because of its excellent clinical and pharmacological action, in addition to its broad spectrum of activity and low resistance rate. However, this drug is not water-soluble, must be administered intravenously and has serious side effects that are moderated with liposomal formulations. Regarding preclinical assays, one of the aspects to be taken into account is the degradation of amphotericin B by light during drug incubation.

Polyenes have shown a prolonged PAFE against *Candida* in some studies, as well as an increase in PAFE with increasing concentrations of the drug (Table 1).

Early studies were performed to determine the in vitro PAFE of amphotericin B against *C. albicans*. Ernst et al. [15] assessed the PAFE of several antifungal drugs by testing two isolates of *C. albicans*: a clinical isolate and the reference isolate from the American Type Culture Collection (ATCC), ATCC 90028. In this work, fungal suspensions were exposed to drug concentrations ranging from 0.125- to 4-times the MIC. Amphotericin B showed prolonged PAFE (>12 h) after exposure for 1 h to concentrations greater than or equal to the MIC of the organism. For that same exposure time and concentrations below the MIC, the PAFE of amphotericin B ranged from 2 to >12 h. In addition, these authors investigated the effect of drug exposure time and observed a prolonged PAFE of >12 h after exposure for only 0.25 h at concentrations above the MIC. These PAFE values were concordant with those of previous work by Turnidge et al. [55] in which the duration of the PAFE of amphotericin B ranged from 0.5–10.4 h for *Candida*. Chryssanthou et al. [4] analyzed the PAFE of amphotericin B, also testing *C. albicans* ATCC 90028, which obtained lower values. Concentrations equal to and above the MIC were used. The PAFE of amphotericin B increased significantly with the increasing concentrations tested. After 0.25 h exposure with concentrations from 1- to 20-times the MIC, the PAFE values ranged from 0.96 h to 10.67 h, whereas after 0.5 h exposure of concentrations between 1- and 10-times, the MIC ranged from 4.04 h to 11.54 h. A third study including *C. albicans* ATCC 90028 analyzed the PAFE of amphotericin B after exposure for 1 h [20]. The results showed a PAFE of 5.3 h after exposure to the drug in a concentration 8-times the MIC. Moreover, a greater effect was detected as the MIC of the tested isolate increased. Thus, results obtained by Ozkutuk et al. [8] indicated that amphotericin B induced lower PAFE values on a *C. albicans* isolate that was inhibited by a minimum concentration of 0.125 mg/L compared to others whose growths were inhibited at concentrations of 0.25 mg/L, 0.50 mg/L and 1 mg/L for all concentrations tested (1-, 2-, 4- and 8-times the MIC). For example, for exposure at the concentration of twice the MIC, the values were for the four isolates 4.66 h, 7.23 h, 14.71 h and 19.45 h, respectively.

Other studies analyzed the PAFE of amphotericin B and nystatin using a larger number of *C. albicans* isolates with different results. For the study of polyene induced PAFE Egusa et al. [56] tested 20 oral isolates of *C. albicans*. These authors observed, after 1 h of exposure with a concentration of 2-times the MIC of the drugs, a PAFE of 8.73 h for amphotericin B and 5.99 h for nystatin. With the same experimental conditions and testing 10 oral isolates of *C. albicans*, Anil et al. [6] obtained similar results for amphotericin B and considerably higher results for nystatin, with mean values of 9.93 h and 12.31 h, respectively. Subsequently, the same group obtained lower PAFE values for both polyenes using a larger number of isolates. Thus, Ellepola et al. [57] analyzed the PAFE of polyenes testing 50 oral isolates of *C. albicans*. The drug concentration was 2-times the MIC and the drug exposure lasted for 1 h. The mean PAFE of the 50 isolates was similar for the two polyenes, with values of 2.18 h for amphotericin B and 2.20 h for nystatin.

The in vitro PAFE of polyenes has been less well studied on other *Candida* species, with some variation in effect compared to that observed on *C. albicans*. Some studies have detected a greater effect on *Candida* species other than *C. albicans* [6,58]. These differences could be related to slight changes in the structure of the different species and to the higher virulence of *C. albicans*, which would allow a faster recovery of growth after transient exposure to drugs. Thus, Ellepola et al. [58] analyzed the effect after 1 h of exposure to nystatin on five oral isolates of six different species. The mean duration of PAFE caused by nystatin was shorter on *C. albicans* (6.85 h) compared to the other species tested, which were *C. glabrata* (8.51 h), *Candida guilliermondii* (8.68 h), *Candida krusei* (11.58 h), *Candida parapsilosis* (15.17 h) and *C. tropicalis* (12.73 h). In this study, in addition to interspecies variations, they found statistically significant intraspecific variation in the PAFE on each of the six *Candida* species examined. Moreover, a significantly longer PAFE on *C. tropicalis* compared to *C. albicans* for both amphotericin B and nystatin, as well as other drugs, were detected by Anil et al. [6]. The results of the assay with 10 oral isolates of *C. tropicalis* after exposure for 1 h to a drug concentration twice the MIC were 12.42 h for amphotericin B and 14.83 h for nystatin, compared with 9.93 h and 12.31 h, respectively, obtained on *C. albicans*. Chryssanthou et al. [4] tested, apart from *C. albicans* ATCC 90028, the reference strains *C. glabrata* ATCC 90030 and *C. krusei* ATCC 6258. An increase in PAFE was also observed at the higher concentrations studied, in addition to some variation in PAFE duration on *C. glabrata* and *C. krusei* strains compared to *C. albicans*. After 0.25 h exposure with concentrations of 5- and 20-times the MIC, the PAFE values were 3.19 h and 5.02 h on *C. glabrata,* while after 0.5 h exposure, the values were 4.18 h and 6.65 h, respectively. In the case of *C. krusei,* the PAFE values at concentrations of 2.5- and 10-times the MIC were 3.33 h and 9.65 h after 0.25 h exposure, and 5.27 h and 14.24 h after 0.5 h exposure, respectively. Samaranayake et al. [59] reported that the most effective antifungal drugs in producing a prolonged PAFE on 14 isolates of *C. glabrata* were the two polyenes, amphotericin B and nystatin, compared to ketoconazole and 5-fluorocytosine. The results indicated that the PAFE values depended on the MIC value and the antifungal drug tested. These values after 1 h of exposure and subsequent drug elimination varied for amphotericin B and nystatin, with significant intraspecific variations in the PAFE values observed. The duration of the PAFE of amphotericin B at concentrations for 1-, 2- and 4-times the MIC ranged from 5.92–10.5 h, 6.42–18.5 h and 8.67–22 h, respectively. On the other hand, the PAFE values of nystatin were much lower compared to the results obtained with amphotericin B, the values ranged from 0 to 9.36 h, 3.60 to 17.83 h and 0 to 13.57 h for 1-, 2- and 4-times the MIC, respectively.

The effect of amphotericin B and nystatin on 20 oral isolates of *C. dubliniensis* after brief exposure resulted in PAFE values of 2.21 h for amphotericin B and 2.17 h for nystatin [10,12]. These results are comparable to those published by the same group on *C. albicans*, a closely related species [57].

Two bloodstream isolates each of *C. guilliermondii*, *Candida kefyr* and *C. lusitaniae* were tested by Di Bonaventura et al. [19]. These authors found that amphotericin B induced significant effect on all of the isolates tested. The effect, as described above, was drug dose-dependent and ranged from 1.3 to 9.4 h, 3.6 to 10 h and 9.2 to 14.9 h at 0.125-, 0.25- and 1-times the MIC, respectively. Results by species were not detailed; however, it is noted that at 4- and 8-times the MIC, amphotericin B produced a PAFE of approximately 13 h on one of the *C. lusitaniae* isolates.

In general, the studies analyze the in vitro effect using short exposure times between 0.5 and 2 h; however, longer times, up to 12 h, have also been tested. The study of Mínguez et al. [44], in addition to using long exposure times (12 h), analyzed the effect of human serum on PAFE. Two isolates of *C. albicans* were tested to determine the effect of amphotericin B after 12 h of exposure. The PAFE lasted up to 4.1 h on one of the isolates at the maximum concentration tested, 1 mg/L. When the experiments were carried out in the presence of 10% human serum, the PAFE values were significantly prolonged. García et al. [18] highlighted the importance of drug concentration and the influence of exposure time on PAFE duration. Amphotericin B-induced PAFE was greater for the longer antifungal exposures. They evaluated the PAFE on *C. albicans* ATCC 10231 and *C. glabrata* ATCC 2001 after exposure for 1.5, 3 and 12 h at drug concentrations of 1-, 4- and 8-times the MIC. PAFE values increased with exposure time, e.g., from 3.5 h to 6.5 h and 8 h for the 4-times the MIC concentration on *C. albicans* with the three exposure times, respectively.

Few studies have assessed the in vitro PAFE of antifungal drug combinations. Oz et al. [3] evaluated the PAFE of caspofungin, voriconazole and amphotericin B, and combinations of caspofungin and voriconazole and caspofungin and amphotericin B. These authors tested the effect on 30 clinical isolates of *C. krusei* because of its known intrinsic resistance to fluconazole and lower susceptibility to other drugs. The concentrations used were 0.25-, 1- and 4-times the MIC of each of them individually and exposure lasted 1 h. The drug that produced the longest PAFE was caspofungin and the combination of caspofungin with amphotericin B at 4-times their MICs resulted in a synergistic interaction against most isolates.

## 4. PAFE of Azoles

Azole antifungal drugs, or azoles, are a heterogeneous group of synthetic fungistatic drugs characterized by a five-atom azole ring linked to other aromatic rings. The azole ring may contain two or three nitrogens and depending on the latter characteristic, the azoles are divided into imidazoles and triazoles. The imidazoles, which have two nitrogens in the main ring structure, include miconazole, ketoconazole and clotrimazole. The triazoles, on the other hand, present three nitrogens in the azole ring and include fluconazole, itraconazole, voriconazole, posaconazole and isavuconazole [1,54].

The azoles act by inhibiting the enzyme 14-α demethylase (Erg11), an important enzyme in ergosterol biosynthesis. This inhibition occurs by complexing the azole with part of the fungal cytochrome P-450. The function of the enzyme is the conversion of lanosterol to ergosterol and its blockage results in an accumulation of toxic precursor molecules, which ultimately leads to a structural and functional alteration of the membrane.

The azoles show, in general terms, a broad spectrum of action, but it depends on each drug. Imidazoles show good activity against yeasts, dermatophytes, endemic fungi and some filamentous fungi. However, their toxicity is higher than that of the triazoles and they are mainly used topically.

As for the triazoles, fluconazole has activity against most *Candida* species, *Cryptococcus*, dermatophytes and endemic fungi, although it is inactive against filamentous fungi. In addition, *C. krusei* shows intrinsic resistance, *C. glabrata* shows reduced sensitivity and secondary resistance is common. Itraconazole, voriconazole, posaconazole and isavuconazole have a broader spectrum, with activity against yeasts and many filamentous fungi.

Ketoconazole is the imidazole whose in vitro PAFE has been most extensively studied in vitro. It has been evaluated mainly against oral *Candida* isolates (Table 2). Testing 10 and 50 isolates of *C. albicans* under similar experimental conditions, with 1 h of exposure at drug concentrations of 2-times the MIC, Anil et al. [6] obtained a PAFE of 1.14 h and Ellepola, et al. [57] of 0.62 h. The study by Anil et al. [6] also included 10 isolates of *C. tropicalis* and obtained a significantly longer PAFE (2.03 h) than on *C. albicans*. Exposure for 1 h at concentrations 3-times the MIC of ketoconazole induced a PAFE of 0.6 h on 20 isolates of *C. dubliniensis* [12]. In contrast to the polyenes evaluated in the study by Samaranayake et al. [59], the PAFE of ketoconazole had a shorter duration for most *C. glabrata* isolates after exposure to the different drug concentrations. Finally, in the study by García et al. [18], ketoconazole was not able to induce significant PAFE against *C. albicans* ATCC 10231 and *C. glabrata* ATCC 2001.

Concerning triazoles, fluconazole is not able to induce PAFE against different *Candida* species in the vast majority of published in vitro studies [6,12,15,18,19,44,57]. Manavathu et al. [20] evaluated the effect on *C. albicans* 90028 of seven drugs, amphotericin B, itraconazole, voriconazole, posaconazole, ravuconazole, and the echinocandins caspofungin and micafungin. Unlike amphotericin B and echinocandins that gave rise to between 5 and 5.6 h PAFEs, triazoles produced a short PAFE (≤0.5 h) and summarized that triazoles and fungistatic drugs produced a short PAFE unlike fungicidal drugs.

Most in vitro PAFE studies with fluconazole have not detected remarkable effects, however, the in vivo PAFE results of Andes et al. [60] demonstrated significant and persistent effects after treatment with this azole. A murine model of disseminated *C. albicans* infection was used to characterize the activity of fluconazole. Treatment with the two studied doses (3.125 and 12.5 mg/kg) significantly flattened *Candida* growth curves, suppressing growth for between 4 and 21 h after serum levels had fallen below the MIC compared to growth in the controls. The study by Maki et al. [52] analyzed in the in vivo model the activity of three azoles, fluconazole, itraconazole and ketoconazole, on disseminated *C. albicans* ATCC 90028 infection. Significant effect of the azoles was also detected in this study. The PAFE detected was similar for all three drugs with values of 10 h for fluconazole, 10.5 h for itraconazole and 11.2 h for ketoconazole. In a murine model of disseminated *C. albicans* infection, Andes et al. [49] characterized the activity of posaconazole. Twelve clinical strains of *C. albicans,* including susceptible and fluconazole-resistant strains, were used. Studies of the PAFE of a single dose demonstrated the suppression of microbial growth after serum levels of posaconazole had fallen below the MIC from 20 h to 30 h. Warn et al. [51] studied the PK and PD of isavuconazole in a murine model of disseminated *C. albicans* candidiasis and detected a higher PAFE value in vivo than in vitro. In the in vitro study, at concentrations of less than or equal to the MIC of isavuconazole, no PAFE was observed, while at concentrations of 2-times the MIC the PAFE was 2 h and at higher concentrations (5-, 10-, 40-, and 100-times the MIC) the PAFE was 5 h. As for the results of the in vivo model, the PAFE was 8.41 h. Andes et al. [47] reported similar results for ravuconazole and for the rest of the triazoles. Ravuconazole suppressed the regrowth of organisms at each of the doses studied. The growth of microorganisms was suppressed for 9.8 and 2.9 h at the 10 and 40 mg/kg doses, respectively.

These studies propose one of the probable reasons why in vivo studies of triazoles demonstrate prolonged PAFE durations and in vitro studies do not. In vivo determinations cannot differentiate between persistent growth suppression due to initial concentrations above the MIC in serum and those potentially due to effects below the MIC [47,49,60].

## 5. PAFE of Echinocandins

The echinocandins are the most recently incorporated group of systemic antifungal drugs, comprising the agents anidulafungin, caspofungin, micafungin and rezafungin, the latter under development [61]. Nowadays, anidulafungin, caspofungin and micafungin are considered the first choice for invasive candidiasis treatment, and they are administered as single daily intravenous doses with minimal side effects [62].

They share a structure based on amphiphilic cyclic hexapeptide with an N-linked acyl-lipid side chain, variable in each of the compounds [54]. The mechanism of action of echinocandins is based on the inhibition of the synthesis of 1,3-β-D-glucan, an essential component of the fungal cell wall responsible for maintaining its stability, which confers a fungicidal effect against *Candida*. Mutations in the *FKS1* and *FKS2* genes cause alterations in the enzyme complex responsible for the synthesis of 1,3-β-D-glucan, which is less susceptible to the activity of echinocandins, leading to an increase in the MICs and a decrease in the therapeutic effect [1,54].

The compounds included in this group have a similar broad spectrum of in vitro and in vivo activity against most *Candida*, although species of the *C. parapsilosis* complex and *C. guilliermondii* present higher MICs.

The echinocandins PAFE length is dependent on the concentration of these antifungal drugs administered and varies between the different *Candida* species (Table 3).

With respect to anidulafungin, the experiments carried out in vitro on *C. albicans* species, revealed a PAFE in the different studies that varied from values >12 h, reported by Ernst et al. [15] and Nguyen et al. [23], after 1 h of exposure to concentrations both below and above the MIC; to values above 33.6 h, and above 37.7 h reported by Gil-Alonso et al. [32] after exposure to anidulafungin for 1 h at 2 mg/L and ≤ 0.5 mg/L. In this latter work, which also analyzed the PAFE on closely related species such as *C. dubliniensis* and *C. africana*, a similar effect (although slightly lower) was detected [32].

On the *C. parapsilosis* complex (including *C. parapsilosis*, *C. metapsilosis* and *C. orthopsilosis*), exposure to anidulafungin (8 mg/L) for 1 h produced a PAFE greater than 42 h, with no differences observed between the different species of the complex [32]. Prolonged PAFE higher than 24 h was also observed in the work by Smith et al. [26] when analyzing one isolate of *C. parapsilosis* and *C. glabrata*. In view of these initial results, these authors increased the time of analysis, and observed prolonged PAFE ranging from >48 h to >144 h. Nguyen et al. [23] also described a PAFE longer than 12 h on *C. parapsilosis*, *C. glabrata* and *C. krusei*.

The PAFE after the exposure to caspofungin has been more variable in the different studies, with values of 2.14 and 2.17 h on *C. albicans* and *C. dubliniensis*, respectively, reported by Ellepola et al. [13], using concentrations 3-the MIC, to values of 5.6 h [20], >12 h [15] and >24 h [21] detected in other works. Gil-Alonso et al. [30] observed effect for 38.41 h on isolates belonging to the *C. albicans* clade, such as *C. albicans*, *C. dubliniensis* and *C. africana*.

The PAFE of caspofungin has also been assessed on other species such as *C. guilliermondii*, *C. kefyr* and *C. lusitaniae.* In the study carried out by Di Bonaventura et al. (2004), PAFE was only detected on one isolate of *C. lusitaniae* [19]. In the case of *C. parapsilosis*, PAFE of 13.93 h [30], >24 h, including *C. parapsilosis* and *C. glabrata* [21], and even >48 h to >144 h on one strain of *C. parapsilosis* and one of *C. glabrata* [26], have been described. In the case of *C. krusei*, a concentration-dependent PAFE was detected with maximum values of >45 h [3].

It is worth highlighting the work by Shields et al. [25], as these authors assessed the PAFE of caspofungin after short periods of exposition to this antifungal agent (5, 15, 30 and 60 min). After exposures for only 5 min at concentrations 4-times the MIC, a PAFE of >24 h on 2 of 4 strains was achieved. In the report of Ernst et al. [15], they also observed that exposure to concentrations above the MIC for 0.25 h resulted in a PAFE of >12 h and that even exposure to concentrations below the MIC for 0.5 h resulted in a PAFE from 0 to 2 h.

As mentioned in Section 2 on methods for PAFE studies, the influence of the medium seems to be a key factor when measuring the PAFE, as can be seen in the work carried out by Kovacs et al. [28], since, although a PAFE was observed after exposure to caspofungin when the experiments were carried out in RPMI, these values decreased when 50% serum was added. Thus, at the minimum concentration tested, 4 mg/L, the PAFE decreased from 4.89–>19.34 to 0–2.27 h in the presence of serum; at the maximum concentration tested, 32 mg/L, the PAFE decreased from 7.65–>19.88 h to 0.24–>18.79 h in the presence of serum. Interestingly, in this work, they analyzed the effect on an echinocandin-resistant *Candida* strain, and no PAFE was observed, irrespective of the medium used.

Variable PAFE duration has also been reported for micafungin. The highest PAFE values were obtained on *C. albicans* or related species, as some studies have observed PAFEs of ≥12 h [24] or even ≥37.5 h after exposure to micafungin at 2 mg/L [29]. In the case of micafungin, the relationship of the presence of serum with a decrease in the time of PAFE was also evident, as for example, in the work of Kardos et al. [31]. Values ranged from 1.5 to 20.1 h when performing the experiments in RPMI, through values ≤1.7 h and even to no PAFE being detected [31]. Experiments against *C. parapsilosis* revealed lower PAFE of micafungin than against *C. albicans*, with values ranging from 2.8–15.7 h [29] or ≥12 h [24].

Micafungin exhibited prolonged PAFE on *C. krusei,* both in experiments carried out in the absence of serum, ≥12 h [24], and in the presence of serum, >19.5 and >20.1 h [63]. In the case of *C. glabrata*, although some authors reported higher values of PAFE, >12 h and >24 h [24,26], experiments in the presence of serum again showed a reduction in the effect with values of 2.6 and 3.4 h [63].

The only in vivo study carried out characterized the PD effect of anidulafungin in a neutropenic murine model [50]. Anidulafungin presented prolonged PAFE ranging from 56 to >96 h on *C. albicans* and from 19 to 67 h on *C. glabrata* infection.

With regard to rezafungin, to date, no PAFE studies have been reported. The appropriate PK characteristics of this new echinocandin, such as its high elimination half-life and tissue penetration, would contribute to its potent antifungal activity and allow for a once-weekly administration schedule, as observed in animal studies [64,65].

## 6. PAFE of 5-Fluorocytosine

5-fluorocytosine is an antifungal drug that interferes with the biosynthesis of nucleic acids. Susceptible cells import the molecule through the enzyme cytosine permease. Inside the fungal cell, 5-fluorocytosine exerts its effect after conversion to its active form, 5-fluorouracil, by cytosine deaminase. The molecules generated by this conversion disrupt multiple cellular processes, including RNA, protein and DNA synthesis.

This drug acts mainly against *Cryptococcus*, *Aspergillus*, *Candida* and some dematiaceous fungi. Fungal resistance to 5-fluorocytosine can be intrinsic or acquired. It is rarely used in monotherapy due to its proven synergism with other agents and to avoid the development of acquired resistance [1,54].

The PAFE of 5-fluorocytosine has been less extensively studied (Table 4). The different studies find a significant PAFE of 5-fluorocytosine of up to more than 10 h [6,18,55,59]. Differences have been observed depending on drug concentration and exposure time. As with other antifungal drugs, some studies have observed a lower effect value on *C. albicans* than on other *Candida* species [6,18].

Regarding the analysis of in vivo PAFE, the study by Andes et al., using the murine model and infection by a clinical isolate of *C. albicans,* showed that the values increased with the dose of 5-fluorocytosine. These values increased from 3.3 to 15.1 h with drug doses between 6.25 and 100 mg/kg [66].

## 7. Mechanisms Involved in PAFE

The mechanism of action of PAFE is unknown, although it has been suggested that many factors could be involved, such as persistence of the drug at the site of action, the time that the microorganism requires to recover the synthesis of ergosterol in the cell membrane, or the synthesis of 1,3-β-D-glucan in the cell wall once exposed to an antifungal drug and withdrawn [23]. The duration of the PAFE and the differences in this parameter among fungal species might be related to the growing features of the cell, the inoculum size, the affinity of the drug for the target or the amount of glucan in the fungal wall, as well as the concentration of the antifungal drug. In this regard, echinocandins are rapidly associated with their target, the 1,3-β-D-glucan synthase, on which they exert a prolonged effect. Alternatively, echinocandins, large lipopeptides, could be rapidly intercalated in the phospholipid bilayer of the membrane of *Candida*, and access its target progressively over time. Moreover, Nguyen et al. [24] observed that micafungin PAFE inhibited the growth of *C. albicans*, *C. glabrata*, *C. krusei* and *C. parapsilosis*, and induced cell wall disturbances detected by electron microscopy that were observable 32 h after drug exposure. These isolates were more susceptible to echinocandins, suggesting the rapid association of these drugs with the target and their maintained activity. Cell wall disturbances induced by micafungin PAFE led to a reduced adherence to buccal epithelial cells and a higher susceptibility to killing by phagocytes. However, other authors propose an additional explanation. Louie et al. [67] suggested that the prolonged residual antifungal activity of caspofungin could be related to the long tissue half-life of caspofungin (59 h vs. serum half-life of 20.5 h), as determined in a murine model of systemic candidiasis.

Regarding differences in PAFE among the three echinocandins, the different lipophilicities of the compounds or the affinity of binding to the target may explain the shorter PAFE of micafungin observed against certain isolates, compared to the PAFE observed for anidulafungin or caspofungin [63].

In contrast to echinocandins, the PAFE of amphotericin B may be explained by the effect of this drug on the fungal cell by physically creating pores. This physical damage makes the effect quick and short and requires only brief exposure to induce a prolonged disabling effect on the fungal cell, as suggested by Manavathu et al. [20]. In addition, several in vitro and in vivo studies have suggested that amphotericin B enhances immune response [68,69], which could contribute to the therapeutical success in in vivo models.

The PAFE of nystatin was determined for oral *C. dubliniensis* isolates, obtaining suppression of growth of 2 h. It was assumed that drug-induced cell wall structure disturbances would be responsible for this PAFE, as well as the suppression of adhesion to epithelial cells and germ tube formation observed [10].

Finally, in vitro studies have evidenced that triazoles do not induce a significant PAFE in *Candida*. However, Warn et al. [51] evaluated the PK and PD of isavuconazole in a murine model of disseminated candidiasis and assessed the in vivo and in vitro PAFE of this newest triazole. These authors obtained in vitro and in vivo PAFEs from 2–5 h and 8.4 h, respectively, suggesting that the persistence of this triazole in the kidney may be linked to the PAFE observed. Significant persistent effects in in vivo PAFE studies for other triazoles, such as fluconazole, were estimated to be from 4–21 h. Different factors are consistent with this in vivo PAFE of triazoles, including the sub-MIC effects, the time for fungal cell to recover from disrupted ergosterol synthesis, and the persistence of the drug at the effect site [47,49].

## 8. Relevance of PAFE in the Design of Dosing Therapeutical Regimens: Clinical Applicability

**Figure 2:**
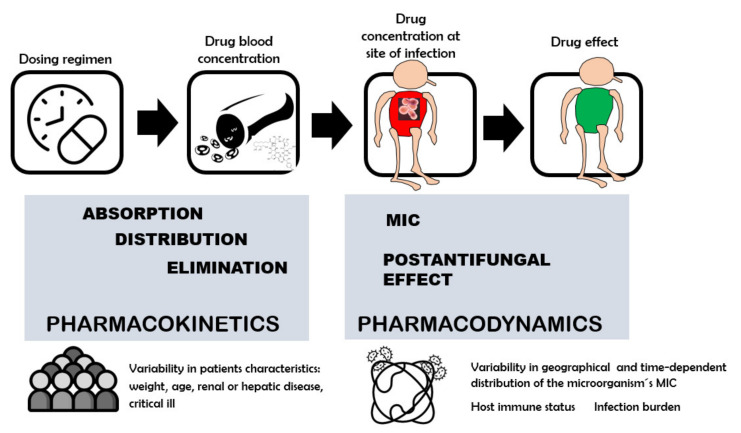
Dose–antifungal response relation. PK and PD properties of antifungal drugs and main sources of variability.

Successful treatment of candidiasis requires the choice of the most suitable antifungal agent and dose regimen [62]. Optimally dosing antifungal therapy is dependent on several factors, such as pathophysiological characteristics of the patients and their immune status, the infecting organism, the site of infection and the PK/PD properties of the antifungal drug (Figure 2) [70,71,72].

PK includes the factors affecting drug absorption, distribution, metabolism and elimination, which determine the evolution of the drug concentration in the body [70]. Considering PK aspects of antifungal therapy in mucocutaneous and invasive/disseminated candidiasis, the site of infection is the extracellular fluid compartment, and, therefore, serum or plasma drug concentrations correlate well with drug concentrations at the site of infection [49]. The antifungal exposure in plasma can be measured as peak plasma concentration (C_max_) and area under the concentration–time curve over a period of 24 h (AUC_0–24h_), PK variables that are dependent on the dose used and the PK properties of each antifungal drug. Additionally, only the unbound drug concentrations are able to access to the fungal target and, thus, are microbiologically active [70,71]. At the site of infection, antifungal drugs act according to their MIC and PAFE [71,73]. In the context of antifungal drugs, the clinical relevance of plasma protein binding changes has been assessed for both triazoles and echinocandins [74,75,76].

Echinocandins are administered as single intravenous daily doses [72]. However, other dosing strategies merit study, such as larger doses less frequently administered with the same cumulative doses as the standard daily divided ones, as suggested by Gumbo et al. [2], Andes et al. [77] and Prépost et al. [78]. Although the clinical evidence that supports the usefulness of larger single echinocandin doses is still very scarce, high single doses of caspofungin and micafungin have been shown to be effective in murine models of candidiasis [2,77,78]. Prepost et al. [78] reported excellent in vivo efficacy of high single doses of caspofungin (40 mg/kg) against echinocandin-resistant isolates. They proposed potential explanations for this higher efficacy, such as the increased unbound drug fraction of caspofungin in patients with invasive candidiasis (7.6% vs. 3.5% in healthy persons). This fact leads to higher free-caspofungin AUC/MIC and C_max_/MIC values than what is recommended. Other possible reasons include the rapid increase and higher peak concentrations following large doses [2,79]. These authors highlighted the importance of free echinocandin penetration into the site of infection related to the clinical outcomes. Additionally, it has been suggested that elevated single doses of caspofungin can be used safely [80,81]. Apart from the PK explanations aforementioned, the persistent effect of echinocandins could also be related to the prolonged PAFE observed, both in vitro and in vivo.

Careful consideration of the factors affecting PK/PD should allow the selection of the most appropriate antifungal drug when treating candidiasis and establishing the dosage with a better risk: benefit ratio in terms of efficacy, safety and development of resistance [73,82]. The interindividual variability in PK or PD processes is one of the main contributors to the variability in the antifungal dose–exposure-response relation [72]. Physiopathological factors associated with interindividual variability of the patient, such as age [83,84], renal disease or replacement therapy [85,86], hepatic disease [87,88], transplantation [89,90] and critical illnesses [91,92,93,94], may be responsible for the interindividual variability of the antifungal PK processes. In particular, critically ill patients have pathophysiological changes that are responsible for antifungal PK alterations, such as organ failure, reduced protein binding, capillary leakage resulting in an altered drug volume of distribution and use of organ support [82]. Moreover, interacting co-medications may result in the variable PK of antifungal drugs [95,96].

When considering the factors responsible for PD variability, antifungal susceptibility rates can vary widely among different *Candida* species [97]. MIC distribution could vary according to whether the species is susceptible, intermediate, susceptible dose-dependent or resistant to the antifungal drug. Interregional, time-dependent or species differences in MIC distribution associated with local epidemiology and resistance patterns [72,98], and factors associated with the variability range of PAFE values summarized in Figure 1, can contribute to the PD variability of the antifungal drug [99]. All these factors that explain the antifungal drug dose–response relationship support the optimization of adequate dosing regimens in clinical practice (Figure 2).

**Figure 3:**
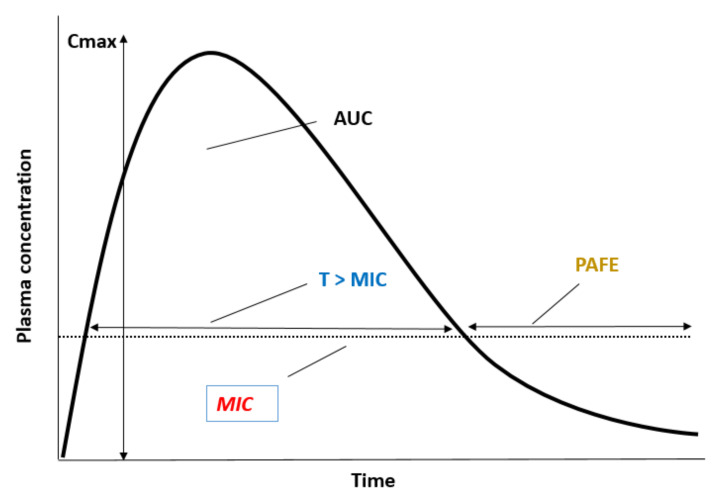
The maximal concentration (C_max_), the ratio of drug area under the concentration–time curve (AUC) to MIC (dotted line) over a 24 h period (AUC_0–24h_/MIC) or the time (expressed as a percentage of the dosing interval) that drug concentrations are expected to exceed the MIC (%T > MIC) as PK/PD index that link the kinetics of antifungal disposition, MIC and PAFE values with the antifungal clinical efficacy.

An in-depth understanding of the relationship between antifungal exposure and clinical response is required to establish useful threshold values for clinical outcome and adverse effects. PK/PD analysis integrates both the PK and PD information for drug to enhance the possibility of success of the antifungal therapy [100,101,102]. The maximal concentration (C_max_), the ratio of drug area under the concentration–time curve (AUC) to MIC over a 24 h period (AUC_0–24h_/MIC) or the time (expressed as a percentage of the dosing interval) that drug concentrations are expected to exceed the MIC (T > MIC) have been used extensively as a PK/PD index that link the kinetics of antimicrobial disposition, MIC and PAFE values and antifungal clinical efficacy. The value of the PK/PD index associated with antimicrobial efficacy varies according to the chosen endpoint, such as stasis, maximal kill or resistance suppression (for preclinical studies), and microbiological or clinical cure (for clinical studies and clinical trials) [70]. Traditionally, C_max_/MIC and AUC_0–24h_/MIC are the optimal drivers that predict the dosing regimens for concentration-dependent antimicrobials, and T > MIC for time-dependent antimicrobials (Figure 3).

The PAFE, as a PD characteristic, can influence the dose–response relationships (Figure 2) [73,103]. Extended PAFE periods allow dosing intervals to be lengthened. For time-dependent antifungal drugs (%T > MIC), the PAFE reduces reliance on concentrations above the MIC and increases relevance of the total drug exposure or AUC/MIC index. For example, triazoles exhibit prolonged in vivo PAFEs, and it has been shown that AUC/MIC is the optimal predictive index [104], whereas flucytosine exhibits limited PAFE, and, in this case, %T > MIC is the optimal efficacy index [66,102]. For polyenes, C_max_/MIC is the PK/PD driver of efficacy. For concentration-dependent drugs, as echinocandins which exhibit a prolonged PAFE, AUC/MIC and C_max_/MIC can both be optimal predictive drivers of efficacy [101,105,106]. Thus, antifungal drugs, depending on whether they are concentration-dependent or time-dependent drugs and whether their PAFE is prolonged or short, have been shown to have different PK/PD clinical efficacy objectives [72].

These PK/PD indexes can be used as a tool to guide the selection of dosing regimens in the studied population, increasing the probability of selecting clinically successful treatments, identifying clinical breakpoints and preventing the emergence of resistance [70,77,98]. Population PK/PD analyses in combination with Monte Carlo simulation [107], incorporating antifungal PK and PD variability, can be combined to computationally estimate the likelihood of a given drug dose to attain a predefined value of a PK/PD target previously defined for antimicrobial drugs [108]. With this clinical translational tool, several clinical objectives can be achieved as dosing regimens expected to have success for a special group of patients, identify drug exposures that may be inadequate, or identify alternative dosing strategies to enhance efficacy (such as dose escalation or increased frequency according PAFE) [108,109,110].

In the case of invasive candidiasis, the clinical validation of PK/PD targets is a final and necessary step to harness the full translational potential of these studies [103]. Although knowledge of the PK/PD of antifungal drugs is increasing due to the many studies in vitro and in experimental animal models being conducted, the practical application of these concepts to individualized dosing at the point of care is still limited. Studies on the PK/PD of 5-fluorocytosine and amphotericin B are quite limited in experimental models concerning invasive candidiasis [102]. Regarding the azole group, fluconazole is the most extensively studied drug using experimental animal models with invasive candidiasis. Its PK/PD index related to efficacy, evaluated in experimental animal models, has been validated in invasive candidiasis through clinical practice. However, data on voriconazole and posaconazole regarding this topic are quite limited. Although a robust PK/PD index of echinocandins have been identified in the laboratory, the translation to the clinical treatment of invasive candidiasis is currently limited [102,106]. Patients with oesophageal candidiasis treated with the standard daily dosing schedule of micafungin showed no difference with a large dosing at prolonged intervals, consistent with animal studies providing AUC/MIC as a predictor of efficacy [77]. The practical application of these concepts to individualized dosing at the point of care is still limited. PAFE as a PD feature and its variability should be considered.

## 9. Conclusions and Proposals for Further Studies

Invasive candidiasis is increasing; highly related to the increase in patients at risk of these infections, such as critically ill patients. These infections are often refractory to the currently available antifungal therapies, as fungal resistance becomes an increasing obstacle to therapy. In order to expand therapeutic options, the selection of the optimal antifungal administration is an important tool in dealing with these infections. In this respect, PAFE has been studied for its potential impact in antifungal drug dosing.

The PAFE attempts to capture the persistent activity of the drug once the isolates are exposed to it, despite the absence of measurable concentrations of the drug in contact with the fungus. In this work, various in vitro and in vivo studies that have determined the PAFE of the main antifungal agents have been compiled. Each model, in vitro and in vivo, has its pros and cons in their ability to assess the postantifungal activity of the drugs, especially when trying to reproduce the results in human infections. Variations in the in vitro/in vivo correlation results may be due to different factors leading to in vitro methods not being able to reproduce the conditions at the site of infection. These factors or conditions include the inoculum, the interaction between drug, pathogen and host immune system, and the PK properties of the drug, among others. In addition, there is no standard method for PAFE determination, hence, standard methods should be defined in order to reduce inter-study variability. Among the issues identified that would require a higher degree of standardization are the medium in which in vitro experiments are carried out. This medium should mimic the conditions of the dynamic environment in the body. Although most PAFE results are obtained in protein-free RPMI medium, several studies have demonstrated that drug exposures in the presence of serum mimic in vivo conditions more reliably. The ideal situation would certainly be to complete and verify in vitro studies with in vivo models, which are closer to human candidiasis. The infection site most commonly used when determining the PAFE in murine models is the kidney. However, in these neutropenic mouse models, when interpreting PAFE results from drugs with a wide tissue distribution, caution should be exercised. In the case of amphotericin B, its high distribution to the kidney is widely known and concentrations reached in kidneys can be much higher than in serum. Hence, it may appear that the antifungal effect continues despite no measurable drug in serum, whereas drug concentrations at the infection site may persist above the MIC and continue to show activity. In these in vivo studies, the state of free drug in the tissues should be verified in order to be able to attribute persistent drug activity to a true PAFE. Additionally, this review has detected a scarcity of PAFE studies in animal models. More in vivo studies should be conducted to explore their translational relevance as a tool that helps to establish the clinical dosing rationale.

After comparing the PAFE of the main groups of antifungal drugs, it can be stated that the echinocandins cause the most prolonged PAFE, followed by polyenes and azoles. In the case of the triazoles, it is worth noting the inconsistency found between in vitro and in vivo studies for the same drug, possibly due to difficulty in differentiating between continued growth suppression related to serum concentrations above MIC and sub-MIC effect. In vivo PAFE studies of triazoles have revealed a longer PAFE. On the other hand, the newest oral and intravenous antifungal drug, ibrexafungerp, should be mentioned. Ibrexafungerp is the first triterpenoid class antifungal agent labeled for the treatment of vulvovaginitis [111]. Although it has been shown that ibrexafungerp displays potent in vitro and in vivo activity against the most clinically relevant species of *Candida* [112], so far, no PAFE studies have been published for this new drug. This semi-synthetic derivative of the terpenoid enfumafungin, acts by inhibiting glucan synthase, decreasing 1,3-β-D-glucan polymers and weakening fungal cell wall [113]. This mechanism of action, similar to that previously described for the echinocandin group, might suggest that ibrexafungerp could also exert a PAFE. However, this should be studied both in vitro and in vivo. In general, such PAFE studies should be carried out for all drugs to be approved by regulatory agencies, given their possible implications for the design of dosing regimens.

On the other hand, considering that there might be species-specific variability in PAFE results, it would be interesting to further evaluate the PAFE of the different antifungal drugs against non-*C. albicans* species, as most PAFE studies have been carried out on *C. albicans*. Some studies have detected a greater PAFE of polyenes on *Candida* species other than *C. albicans*. These differences could be explained by slight changes in the structure of the different species and to the higher virulence of *C. albicans*, which would allow a faster recovery of growth after short exposure to drugs. On the other hand, differences in methodological issues among studies, such as the drug concentrations used, preclude species-dependent PAFE comparisons among studies. Moreover, PAFE has been studied, but not extensively, for fungi other than *Candida*, such as *Aspergillus*, other filamentous fungi and zygomizetes. As remarked in those studies, PAFE appears to be dependent on several factors, such as the concentration of the drug and class of drug, exposure time or media used [114,115,116].

The PAFE, as an exposure–response factor, can impact on the PD relationships. 5-Fluorocytosine exhibits limited or short PAFE and T > MIC is the PK/PD index best related to efficacy, whereas triazoles show prolonged in vivo PAFE and concentration-independent action, suggesting that AUC/MIC is the optimal predictive driver of efficacy. In contrast, for polyenes, C_max_/MIC is the PK/PD driver of efficacy. For echinocandins, with concentration-dependent activity and prolonged PAFE, both C_max_/MIC and AUC/MIC are optimal predictors of efficacy. Additionally, the dosing intervals impact on the treatment efficacy and can determine the PK/PD index. It has been demonstrated that when higher doses are administered with larger dosing intervals and treatment outcome improves, then the C_max_/MIC is the optimal efficacy index. When treatment effect is similar among several dosing intervals, the AUC/MIC index is the predictive index. From a clinical point of view, this could have applicability for echinocandins, concentration-dependent and prolonged PAFE drugs. When the standard daily dosing regimen of micafungin was compared to an extended interval large dose regimen in patients with esophageal candidiasis, no statistically different outcomes were obtained between both dosing schedules, in agreement with animal studies providing AUC/MIC as the efficacy driver.

In conclusion, PAFE is a relevant exposure–response variable to understand and optimize antifungal efficacy in vivo, and it should be taken into account in therapeutic decisions related to dosage regimens. However, further in vivo studies are warranted, including experiments in animal models of candidiasis. These could be taken as a starting point for the study of extended interval dosing regimens, as is the case of echinocandins for treating mycoses.

## Figures and Tables

**Table 1:** PAFE in vitro of polyenes.

Antifungal	Species (Strains)	MIC (mg/L)	Exposure	Concentration	PAFE (h)	Methodology *	Reference
Amphotericin B	*C. albicans* (2)	0.25–1	12 h	0.5, 1 mg/L	0.9–2.5, 2.4–4.1	CC, bYNBg	[44]
Amphotericin B	*C. albicans* (12)	0.19	1 h	2 × MIC	4.69–13.44	OD, RPMI	[56]
Amphotericin B	*C. albicans* (2)	0.5–1	1 h	0.125–4 × MIC	2–>12	CC, RPMI	[15]
Amphotericin B	*C. albicans* (10)	0.19–0.38	1 h	2 × MIC	9.93	OD, RPMI	[6]
Amphotericin B	*C. albicans* (1)	0.5	0.25–0.5 h	1, 2.5, 5, 10, 20 × MIC	0.96–4.04, 2.54–7.68, 3.92–9.13, 7.67–11.54, 10.67	CO_2_, RPMI	[4]
Amphotericin B	*C. albicans* (1)	1	1.5–12 h	1, 4, 8 × MIC	0.8–4.9, 3.5–8.0, 4.6–12	CC, bYNBg	[18]
Amphotericin B	*C. albicans* (1)	0.125	1 h	1–16 mg/L	5.3	CC, RPMI	[20]
Amphotericin B	*C. albicans* (1)	0.125–1	1.5 h	1, 2, 4, 8 × MIC	0.54–16.35, 3.48–19.31, 9.11–19.86, 16.25–21.54	OD, RPMI	[8]
Amphotericin B	*C. albicans* (50)	0.004–0.19	1 h	2 × MIC	2.18	OD, RPMI	[57]
Amphotericin B	*C. dubliniensis* (20)	0.002–0.125	1 h	3 × MIC	1.92–2.41	OD, RPMI	[12]
Amphotericin B	*C. glabrata* (1)	0.5	0.25 h	5, 20 × MIC	3.19, 5.02	CO_2_, RPMI	[4]
Amphotericin B	*C. glabrata* (1)	0.5	0.5 h	2.5, 10 × MIC	4.18, 6.65	CO_2_, RPMI	[4]
Amphotericin B	*C. glabrata*	0.5	1.5–12 h	1, 4, 8 × MIC	1.3–5.3, 3.5–8.6, 4.8–13	CC, bYNBg	[18]
Amphotericin B	*C. glabrata* (14)	0.256–0.512	1 h	1, 2, 4 xMIC	5.92–10.50, 6.42–18.50, 8.67–22.00	OD, RPMI	[59]
Amphotericin B	*C. guilliermondii* (2), *C. kefyr* (2), *C. lusitaniae* (2)	0.5–4	1 h	0.125, 0.25, 1 × MIC	1.3–9.4, 3.6–10, 9.2–14.9	CC, RPMI	[19]
Amphotericin B	*C. krusei* (1)	0.5	0.25 h	5, 20 × MIC	3.33, 9.65	CO_2_, RPMI	[4]
Amphotericin B	*C. krusei* (1)	0.5	0.5 h	2.5, 10 × MIC	5.27, 14.24	CO_2_, RPMI	[4]
Amphotericin B	*C. tropicalis* (10)	0.25–0.38	1 h	2 × MIC	12.42	OD, RPMI	[6]
Nystatin	*C. albicans* (5)	0.78–1.56	1 h	1 × MIC	6.85	OD, RPMI	[58]
Nystatin	*C. albicans* (12)	0.78–1.56	1 h	2 × MIC	1.91–7.99	OD, RPMI	[56]
Nystatin	*C. albicans* (10)	0.78–1.56	1 h	2 × MIC	12.31	OD, RPMI	[6]
Nystatin	*C. albicans* (50)	0.78–1.56	1 h	2 × MIC	2.20	OD, RPMI	[57]
Nystatin	*C. dubliniensis* (20)	0.09–0.78	1 h	3 × MIC	1.92–2.41	OD, RPMI	[10]
Nystatin	*C. glabrata* (5)	0.78–1.56	1 h	1 × MIC	8.51	OD, RPMI	[58]
Nystatin	*C. glabrata* (14)	0.39–1.56	1 h	1, 2, 4 × MIC	0–9.36, 3.60–17.83, 0–13.57	OD, RPMI	[59]
Nystatin	*C. guilliermondii* (5)	0.39–0.78	1 h	1 × MIC	8.68	OD, RPMI	[58]
Nystatin	*C. krusei* (5)	3.12	1 h	1 × MIC	11.58	OD, RPMI	[58]
Nystatin	*C. parapsilosis* (5)	1.56–3.12	1 h	1 × MIC	15.17	OD, RPMI	[58]
Nystatin	*C. tropicalis* (5)	1.56–3.12	1 h	1 × MIC	12.73	OD, RPMI	[58]
Nystatin	*C. tropicalis* (10)	0.78	1 h	2 × MIC	14.83	OD, RPMI	[6]

* The methodology for analyzing the PAFE: by determining the turbidity of the different samples, measuring the optical density (OD) or counting colonies (CC). Culture medium: RPMI 1640 (Roswell Park Memorial Institute) and bYNBg (Yeast Nitrogen Base-Glucose, modified liquid medium of Shadomy).

**Table 2:** PAFE in vitro of azoles.

Antifungal	Species (Strains)	MIC (mg/L)	Exposure	Concentration	PAFE (h)	Methodology *	Reference
Fluconazole	*C. albicans* (2)	2–4	12 h	4, 8 mg/L	No measurable	CC, bYNBg	[44]
Fluconazole	*C. albicans* (2)	0.25	1 h	1, 2, 4 × MIC	No measurable	CC, RPMI	[15]
Fluconazole	*C. albicans* (10)	0.125–0.38	1 h	2 × MIC	No measurable	OD, RPMI	[6]
Fluconazole	*C. albicans* (1)	4	1.5–12 h	1, 4, 8 × MIC	No measurable	CC, bYNBg	[18]
Fluconazole	*C. albicans* (50)	0.047–0.125	1 h	2 × MIC	No measurable	OD, RPMI	[57]
Fluconazole	*C. dubliniensis* (20)	0.016–0.38	1 h	3 × MIC	No measurable	OD, RPMI	[12]
Fluconazole	*C. guilliermondii* (2), *C. kefyr* (2), *C. lusitaniae* (2)	0.12–2	1 h	0.125–8 × MIC	No measurable	CC, RPMI	[19]
Fluconazole	*C. tropicalis* (10)	0.25–0.50	1 h	2 × MIC	No measurable	OD, RPMI	[6]
Itraconazole, Voriconazole, Posaconazole, Ravuconazole	*C. albicans* (1)	0.06–0.25	1 h	1–16 mg/L	≤0.5	CC, RPMI	[20]
Ketoconazole	*C. albicans* (10)	0.012–0.016	1 h	2 × MIC	1.14	OD, RPMI	[6]
Ketoconazole	*C. albicans* (1)	1	1.5–12 h	1, 4, 8 × MIC	No measurable	CC, bYNBg	[18]
Ketoconazole	*C. albicans* (50)	0.004–0.032	1 h	2 × MIC	0.62	OD, RPMI	[57]
Ketoconazole	*C. dubliniensis* (20)	0.002–0.012	1 h	3 × MIC	0.50–0.75	OD, RPMI	[12]
Ketoconazole	*C. glabrata* (1)	1	1.5–12 h	1, 4, 8 × MIC	No measurable, 0–0.3	CC, bYNBg	[18]
Ketoconazole	*C. glabrata* (14)	100–520	1 h	1, 2, 4 xMIC	1.8–5.72, 1.04–5.54, 0.69–5.7	OD, RPMI	[59]
Ketoconazole	*C. tropicalis* (10)	0.064–0.125	1 h	2 × MIC	2.03	OD, RPMI	[6]
Voriconazole	*C. guilliermondii* (2), *C. kefyr* (2), *C. lusitaniae* (2)	0.12–1	1 h	0.125–8 × MIC	No measurable	CC, RPMI	[19]

* The methodology for analyzing the PAFE: by determining the turbidity of the different samples, measuring the optical density (OD) or counting of colonies (CC). Culture medium: RPMI (Roswell Park Memorial Institute) and bYNBg (Yeast Nitrogen Base-Glucose, modified liquid medium of Shadomy).

**Table 3:** PAFE in vitro of echinocandins.

Antifungal	Species (Strains)	MIC (mg/L)	Exposure	Concentration	PAFE (h)	Methodology *	Reference
Anidulafungin	*C. africana* (2)	0.003–0.006	1 h	0.12, 0.5, 2 mg/L	0.7–2.8, 2–>37.7, 36.6–>37.7	CC, RPMI	[32]
Anidulafungin	*C. albicans* (2)	0.015	1 h	0.125–4 × MIC	>12	CC, RPMI	[15]
Anidulafungin	*C. albicans* (4)	0.008–0.03	1 h	1, 4, 16 × MIC	≥12	CC, RPMI	[23]
Anidulafungin	*C. albicans* (7)	0.003–0.006	1 h	0.12, 0.5, 2 mg/L	0–>43, 0–>42, 39, 1–>44	CC, RPMI	[32]
Anidulafungin	*C. dubliniensis* (5)	0.003–0.006	1 h	0.12, 0.5, 2 mg/L	0. 0–>42, 18–>44	CC, RPMI	[32]
Anidulafungin	*C. glabrata* (3)	0.03–0.06	1 h	1, 4, 16 × MIC	≥12	CC, RPMI	[23]
Anidulafungin	*C. glabrata* (2)	0.06	1 h	0.5, 2, 16 × MIC	>24	CC, RPMI	[26]
Anidulafungin	*C. krusei* (2)	0.03–0.06	1 h	1, 4, 16 × MIC	≥12	CC, RPMI	[23]
Anidulafungin	*C. metapsilosis* (2)	1	1 h	0.25, 2, 8 mg/L	0, 0–2, >24	CC, RPMI	[32]
Anidulafungin	*C. orthopsilosis* (2)	1	1 h	0.25, 2, 8 mg/L	0, 0–2, 42–>44	CC, RPMI	[32]
Anidulafungin	*C. parapsilosis* (2)	0.5, 1	1 h	0.5, 2, 16 × MIC	9, 17, >24, >24	CC, RPMI	[26]
Anidulafungin	*C. parapsilosis* (3)	1.0–2.0	1 h	0.25, 2, 8 mg/L	0, 0–5.7, 5.2–42	CC, RPMI	[32]
Anidulafungin	*C. parapsilosis* (3)	1.0–2.0	1 h	1, 4, 16 × MIC	≥12	CC, RPMI	[23]
Caspofungin	*C. africana* (2)	0.5	1 h	0.12, 0.5, 2 mg/L	0–0.7, 0–0.8, 13.5–37.7	CC, RPMI	[30]
Caspofungin	*C. albicans* (2)	0.03	1 h	0.125–4 × MIC	0–>12	CC, RPMI	[15]
Caspofungin	*C. albicans* (1)	0.03	1 h	0.25 mg/L	5.6	CC, RPMI	[20]
Caspofungin	*C. albicans* (4)	0.03–0.25	1 h	1, 4, 16 × MIC	>24	CC, RPMI	[21]
Caspofungin	*C. albicans* (5)	0.03–0.125	5 min	0.25, 1, 8 mg/L	1.4–>24, 1.4–>24, 1.7–3.6	CC, RPMI	[25]
Caspofungin	*C. albicans* (5)	0.03–0.125	15 min	0.25, 1, 8 mg/L	0.09–1.8, 1.2–2.9, 0.8–2.7	CC, RPMI	[25]
Caspofungin	*C. albicans* (5)	0.03–0.125	30 min	0.25, 1, 8 mg/L	0.07–1.9, 0.5–2.1, 0.8–2.5	CC, RPMI	[25]
Caspofungin	*C. albicans* (5)	0.03–0.125	60 min	0.25, 1, 8 mg/L	1.2–>24, 0.02–>24, 1.2–3.0	CC, RPMI	[25]
Caspofungin	*C. albicans* (5)	0.015–3.0	1 h	4, 16, 32 mg/L	4.9–>19.3, 13.8–>19.9, 7.7–>19.9	CC, RPMI	[28]
Caspofungin	*C. albicans* (5)	0.125–0.5	1 h	4, 16, 32 mg/L	0–2.3, 0.3–10.1, 0.2–>18.8	CC, RPMI + 50% serum	[28]
Caspofungin	*C. albicans* (1)	4	1 h	4, 16, 32 mg/L	0	CC, RPMI	[28]
Caspofungin	*C. albicans* (1)	>32	1 h	4, 16, 32 mg/L	0	CC, RPMI + 50% serum	[28]
Caspofungin	*C. albicans* (7)	0.25–0.5	1 h	0.12, 0.5, 2 mg/L	0–2.9, 0–2.3, >39.5–>44	CC, RPMI	[30]
Caspofungin	*C. albicans* (20)	0.004–0.125	1 h	3 × MIC	2.14	OD, RPMI	[13]
Caspofungin	*C. dubliniensis* (20)	0.003–0.19	1 h	3 × MIC	2.17	OD, RPMI	[13]
Caspofungin	*C. dubliniensis* (5)	0.25–0.5	1 h	0.12, 0.5, 2 mg/L	0, 0, 20–>42	CC, RPMI	[30]
Caspofungin	*C. glabrata* (2)	0.25	1 h	1, 4, 16 × MIC	>24	CC, RPMI	[21]
Caspofungin	*C. glabrata* (2)	0.5	1 h	0.5, 2, 16 × MIC	>24, >24, >24	CC, RPMI	[25]
Caspofungin	*C. guillermondii* (2)	128	1 h	4, 8 × MIC	No measurable	CC, RPMI	[19]
Caspofungin	*C. kefyr* (2)	0.25	1 h	4, 8 × MIC	No measurable	CC, RPMI	[19]
Caspofungin	*C. krusei* (30)	0.25–0.125	1 h	0.25, 1.0, 4.0 × MIC	12->45, 10–>45, 20->45	CC, RPMI	[3]
Caspofungin	*C. lusitaniae* (2)	0.5	1 h	4, 8 × MIC	**	CC, RPMI	[19]
Caspofungin	*C. metapsilosis* (2)	1–2	1 h	0.25, 2, 8 mg/L	0, 0, 6.6–>42	CC, RPMI	[30]
Caspofungin	*C. orthopsilosis* (2)	1–2	1 h	0.25, 2, 8 mg/L	0, 0–2, 9–20	CC, RPMI	[30]
Caspofungin	*C. parapsilosis* (2)	0.06–0.5	1 h	1, 4, 16 × MIC	>24	CC, RPMI	[21]
Caspofungin	*C. parapsilosis* (2)	1–0.5	1 h	0.5, 2, 16 × MIC	12, 17, >24, >24	CC, RPMI	[25]
Caspofungin	*C parapsilosis* (3)	1–2	1 h	0.25, 2, 8 mg/L	0, 0, 3.7–11.6	CC, RPMI	[30]
Micafungin	*C. africana* (2)	0.06–0.12	1 h	0.12, 0.5, 2 mg/L	0 h, 0–3 h, >37.5->37.7	CC, RPMI	[29]
Micafungin	*C. africana* (3)	0.015	1 h	4, 16, 32 mg/L	9.7->19.2, >19.2–19.9, >19.20–>20.1	CC, RPMI	[31]
Micafungin	*C. albicans* (4)	0.0312–0.125	1 h	0.25, 1, 4 × MIC	0–0.5,−0.30–4.7, 0.90–>16.6	CC, RPMI	[63]
Micafungin	*C. albicans* (1)	0.125	1 h	0.25 mg/L	5.0	CC, RPMI	[20]
Micafungin	*C. albicans* (4)	0.008–0.125	1 h	1, 4, 16 × MIC	≥ 12	CC, RPMI	[24]
Micafungin	*C. albicans* (7)	0.12–0.25	1 h	0.12, 0.5, 2 mg/L	0–2.4, 00–>43, >39.50–>44	CC, RPMI	[29]
Micafungin	*C. albicans* (3)	0.03–1	1 h	4, 16, 32 mg/L	1.50–>19.3, >18.20–>19.4, >18.2–>19.4	CC, RPMI	[31]
Micafungin	*C. dubliniensis* (5)	0.06–0.25	1 h	0.12, 0.5, 2 mg/L	0 h, 0–20, 42–>44	CC, RPMI	[29]
Micafungin	*C. dubliniensis* (4)	0.015–0.63	1 h	4, 16, 32 mg/L	>15.9–> 18.5, >15.9–19.9, >15.9–18.5	CC, RPMI	[31]
Micafungin	*C. glabrata* (2)	0.0156–0.0625	1 h	0.25, 1, 4 × MIC	019, 0.09, 0.12, 0.45, 3.4, 2.6	CC, RPMI	[63]
Micafungin	*C. glabrata* (2)	0.015–0.125	1 h	1, 4, 16 × MIC	≥12	CC, RPMI	[24]
Micafungin	*C. glabrata* (2)	0.03	1 h	0.5, 2, 16 × MIC	0.3–0.8, 7, > 24, >24	CC, RPMI	[26]
Micafungin	*C. krusei* (2)	0.5	1 h	0.25, 1, 4 × MIC	2.4–4.1, ≥4.5, ≥19.5	CC, RPMI	[63]
Micafungin	*C. krusei* (2)	0.03–0.06	1 h	1, 4, 16 × MIC	≥ 12	CC, RPMI	[24]
Micafungin	*C. metapsilosis* (2)	2	1 h	0.25, 2, 8 mg/L	0, 0, 5.4–9.3	CC, RPMI	[29]
Micafungin	*C. orthopsilosis* (2)	1	1 h	0.25, 2, 8 mg/L	0–2, 0–2, 3.8–11	CC, RPMI	[29]
Micafungin	*C. parapsilosis* (2)	0.5–1	1 h	1, 4, 16 × MIC	≥12	CC, RPMI	[24]
Micafungin	*C. parapsilosis* (2)	1	1 h	0.5, 2, 16 × MIC	0.2, 3, 11, 10, >24	CC, RPMI	[26]
Micafungin	*C parapsilosis* (3)	1–2	1 h	0.25, 2, 8 mg/L	0, 0, 5.3–15.7	CC, RPMI	[29]
Micafungin	*C. tropicalis* (2)	0.5	1 h	0.25, 1, 4 × MIC	<0.2, 5, 0.4, ≥11.6, 2.6	CC, RPMI	[63]

* The methodology for analyzing the PAFE: by determining the turbidity of the different samples, measuring the optical density (OD) or counting of colonies (CC). Culture medium: RPMI (Roswell Park Memorial Institute) and bYNBg (Yeast Nitrogen Base-Glucose, modified liquid medium of Shadomy). ** Measurable PAFE in 1 isolate regardless of concentration; data not shown.

**Table 4:** PAFE in vitro of 5-fluorocytosine.

Antifungal	Species (Strains)	MIC (mg/L)	Exposure	Concentration	PAFE (h)	Methodology *	Reference
5-Fluorocytosine	*C. albicans* (10)	0.25–0.50	1 h	2 × MIC	2.37	OD, RPMI	[6]
5-Fluorocytosine	*C. albicans* (1)	0.5	1.5–12 h	1, 4, 8 × MIC	0.6–2.6, 2.2–5.2, 4.1–6.8	CC, bYNBg	[18]
5-Fluorocytosine	*C. glabrata* (1)	0.0625	1.5–12 h	1, 4, 8 × MIC	1–3, 3–6.2, 4.8–10.8	CC, bYNBg	[18]
5-Fluorocytosine	*C. glabrata* (14)	0.0008–0.025	1 h	1, 2, 4 × MIC	2.2–9.5, 2.5–16.6, 2.8–17.5	OD, RPMI	[59]
5-Fluorocytosine	*C. tropicalis* (10)	0.094–0.125	1 h	2 × MIC	4.41	OD, RPMI	[6]

* The methodology for analyzing the PAFE: by determining the turbidity of the different samples, measuring the optical density (OD) or counting of colonies (CC). Culture medium: RPMI (Roswell Park Memorial Institute) and bYNBg (Yeast Nitrogen Base-Glucose, modified liquid medium of Shadomy).

## Data Availability

Not applicable.
